# Smoking is associated with the concurrent presence of multiple autoantibodies in rheumatoid arthritis rather than with anti-citrullinated protein antibodies per se: a multicenter cohort study

**DOI:** 10.1186/s13075-016-1177-9

**Published:** 2016-12-01

**Authors:** Tineke J. van Wesemael, Sofia Ajeganova, Jennifer Humphreys, Chikashi Terao, Ammar Muhammad, Deborah P. M. Symmons, Alex J. MacGregor, Ingiäld Hafström, Leendert A. Trouw, Annette H. M. van der Helm-van Mil, Tom W. J. Huizinga, Tsuneyo Mimori, René E. M. Toes, Fumihiko Matsuda, Björn Svensson, Suzanne M. M. Verstappen, Diane van der Woude

**Affiliations:** 1 Department of Rheumatology C1-R, Leiden University Medical Center, Albinusdreef 2, PO Box 9600, 2300 RC Leiden, The Netherlands; 2 Arthritis Research UK Centre for Epidemiology, University of Manchester, Manchester, UK; 3 Center for Genomic Medicine, Kyoto University Graduate School of Medicine, Kyoto, Japan; 4 Center for the Promotion of Interdisciplinary Education and Research, Kyoto University Graduate School of Medicine, Kyoto, Japan; 5 Division of Rheumatology, Immunology, and Allergy, Brigham and Women’s Hospital, Harvard Medical School, Boston, MA USA; 6 Division of Genetics, Brigham and Women’s Hospital, Harvard Medical School, Boston, MA USA; 7 Program in Medical and Population Genetics, Broad Institute, Cambridge, MA USA; 8 NIHR Manchester Musculoskeletal Biomedical Research Unit, Central Manchester University Hospitals NHS Foundation Trust, Manchester, UK; 9 Norwich Medical School, University of East Anglia, Norwich, UK; 10 Rheumatology Unit, Department of Medicine, Karolinska Institutet at Karolinska University Hospital Huddinge, Stockholm, Sweden; 11 Department of Rheumatology and Clinical Immunology, Kyoto University Graduate School of Medicine, Kyoto, Japan; 12 Department of Clinical Sciences, Section of Rheumatology, Lund University, Lund, Sweden

**Keywords:** Rheumatoid arthritis, Smoking, Anti-citrullinated protein antibodies, Rheumatoid factor, Anti-carbamylated protein antibodies, Risk factor

## Abstract

**Background:**

The contribution of smoking to rheumatoid arthritis (RA) is hypothesized to be mediated through formation of anti-citrullinated protein antibodies (ACPA). In RA, however, autoantibodies such as ACPA, rheumatoid factor (RF), and anti-carbamylated protein antibodies (anti-CarP) often occur together, and it is thus unclear whether smoking is specifically associated with some autoantibodies rather than others. We therefore investigated whether smoking is only associated with ACPA or with the presence of multiple RA-related autoantibodies.

**Methods:**

A population-based Japanese cohort (*n* = 9575) was used to investigate the association of smoking with RF and anti-cyclic citrullinated peptide antibodies (anti-CCP2) in individuals without RA. Furthermore, RA patients fulfilling the 1987 criteria from three early arthritis cohorts from the Netherlands (*n* = 678), the United Kingdom (*n* = 761), and Sweden (*n* = 795) were used. Data on smoking, RF, anti-CCP2, and anti-CarP were available. A total score of autoantibodies was calculated, and odds ratios (ORs) and 95% confidence intervals (95% CIs) were calculated by logistic regression.

**Results:**

In the population-based non-RA cohort, no association was found between smoking and one autoantibody (RF or anti-CCP2), but smoking was associated with double-autoantibody positivity (OR 2.95, 95% CI 1.32–6.58).

In RA patients, there was no association between smoking and the presence of one autoantibody (OR 0.99, 95% CI 0.78–1.26), but smoking was associated with double-autoantibody positivity (OR 1.32, 95% CI 1.04–1.68) and triple-autoantibody positivity (OR 2.05, 95% CI 1.53–2.73).

**Conclusions:**

Smoking is associated with the concurrent presence of multiple RA-associated autoantibodies rather than just ACPA. This indicates that smoking is a risk factor for breaking tolerance to multiple autoantigens in RA.

**Electronic supplementary material:**

The online version of this article (doi:10.1186/s13075-016-1177-9) contains supplementary material, which is available to authorized users.

## Background

Rheumatoid arthritis (RA) is a systemic autoimmune disease, characterized by synovial inflammation and joint destruction [1]. Current concepts of the pathophysiology of RA are based on associations between RA and risk factors [2]. Among the genetic risk factors, the human leukocyte antigens (HLA)-DRB1 locus with the shared epitope (SE) alleles is associated with the largest effect size [3]. The most prominent environmental risk factor for RA is smoking. Autoantibodies such as rheumatoid factor (RF) are present in the majority of RA patients and are known to develop years before disease onset [4, 5]. Smoking was originally described to be associated with RF-positive RA [6–8]. The discovery of anti-citrullinated protein antibodies (ACPA) led to a paradigm shift in the field of RA risk factor analysis, because many known predisposing factors were found to be specifically associated with ACPA-positive RA rather than ACPA-negative RA [9–11]. This particularly applies to the HLA SE alleles and smoking, for which a gene–environment interaction has been described for ACPA-positive RA [12, 13]. Because of this striking finding, smoking was incorporated into a now widely adopted pathophysiological model explaining the processes underlying ACPA formation [14]. According to this model, smoking exerts its influence many years before disease onset by causing citrullination of proteins in the lungs. An autoimmune response against these proteins then leads to the production of ACPA in HLA SE-positive individuals.

While this hypothesis is based on a specific link between smoking, HLA SE alleles, and ACPA, smoking has also been reported to be associated with other RA-related autoantibodies. In mice and humans with chronic lung disease, smoking has been reported to induce production of RF rather than ACPA [15]. Furthermore, recent research in a population-based cohort in Japan revealed in non-RA healthy individuals that there was a dose-dependent association of smoking not only with high levels of ACPA but also with high levels of RF [16].

This raised the question whether the association of smoking with RA is limited to ACPA-positive RA, or whether smoking is associated with other autoantibodies and/or multiple autoantibodies as well.

To address this question, we first investigated the association of smoking with RF and anti-CCP2 in a population-based cohort of healthy individuals because smoking presumably exerts its influence years before disease onset [4, 5, 17]. Next, we investigated the association of smoking and anti-CCP2, IgM-RF, and anti-carbamylated protein antibodies (anti-CarP) in RA patients from three independent cohorts. Anti-CarP is a more recently described autoantibody present in established RA as well as in the pre-RA phases [5, 18–23]. Finally, we also studied whether smoking might be associated with the breadth of the autoimmune response, reflected by the number of autoantibodies. Furthermore, we looked at anti-nuclear antibodies (ANA) to see whether the association between smoking and autoantibodies is specific for RA-related autoantibodies or exists for autoantibodies in general.

## Methods

### Populations

Data on non-RA subjects were derived from the Japanese Nagahama Prospective Genome Cohort for Comprehensive Human Bioscience (Nagahama study) [16]. Volunteers without autoimmune disease from Nagahama city were recruited from 2008 to 2010.

Original individual patient data of RA patients were used from three independent early arthritis cohorts from the United Kingdom (*n* = 678), the Netherlands (*n* = 769), and Sweden (*n* = 795). The UK dataset originated from the Norfolk Arthritis Register (NOAR) [24]. Data on Dutch RA patients were provided from the Leiden Early Arthritis Clinic (EAC) [25]. The Swedish data originated from the Better Anti‐Rheumatic Farmaco-Therapy (BARFOT) cohort [26]. Inclusion criteria and details of these cohorts have been described elsewhere [24–26]. From each cohort, patients who fulfilled the 1987 criteria for RA with disease duration shorter than 2 years and for whom information on smoking and all three autoantibodies under study was available were included in the current analysis.

The protocol of each cohort was approved by the relevant local ethics committee and all participants provided written informed consent.

### Smoking

Data concerning smoking were collected at baseline. In the NOAR, data on smoking history were obtained by designated study nurses. In the BARFOT, each patient’s smoking history was assessed by the rheumatologist. Information regarding smoking history in the EAC and the Nagahama study was obtained using a questionnaire.

Smoking was defined as smoking of cigarettes. In all cohorts, distinction was made between never smoking and ever smoking. In addition, for the NOAR, the EAC, and the BARFOT, information on former smoking versus current smoking was available. No data on pack-years were available.

### Genotyping

For the majority of patients in the EAC (*n* = 652) and the NOAR (*n* = 544), HLA-DRB1 genotyping data were available. DNA was collected at baseline for genotyping by polymerase chain reaction and hybridization to sequence-specific oligonucleotides as described previously [27]. The HLA-DRB1*01:01, HLA-DRB1*01:02, HLA-DRB1*04:01, HLA-DRB1*04:04, HLA-DRB1*04:05, HLA-DRB1*04:08, HLA-DRB1*10:01, and HLA-DRB1*14:02 alleles were classified as the SE alleles.

### Serological measurements

All measurements were performed in serum samples collected at baseline, except for anti-CarP in the NOAR which was measured in serum taken within the first year of follow-up. A commercial enzyme-linked immunosorbent assay was used to measure anti-cyclic citrullinated peptide antibodies (anti-CCP2); Diastat (Axis-Shield) for the NOAR; MesaCup for the Nagahama study; and Eurodiagnostica for the EAC and the BARFOT. Cutoff values were specified by the manufacturer.

IgM-RF was measured by commercial tests: ELISA in the EAC, agglutination test (SERODIA-RA) in the BARFOT, and latex turbidimetric immunoassay in the NOAR and the Nagahama study. Cutoff values were specified by the manufacturer.

IgG-anti-CarP antibodies were measured in Leiden for all cohorts with carbamylated fetal calf serum (FCS) as antigen using in-house ELISAs. The cutoff value for a positive response was established as the mean plus two times the standard deviation (SD) of the specific anti-CarP reactivity of healthy controls [18].

ANA were determined by indirect immunofluorescence on Hep-2 cells. Titers above 1:40 were considered positive in the EAC. Titers above 1:80 were considered positive in the Nagahama study [28].

Furthermore, in a random selection of patients from the EAC cohort (*n* = 386), serum total IgG levels were measured using the Human IgG-ELISA Quantitation Set (Bethyl Laboratories, USA) according to the manufacturer’s instructions.

### Statistical analysis

The association between smoking (ever versus never) as the explanatory variable and the presence of autoantibodies as the outcome (or dependent) variable was assessed by logistic regression analysis, used to calculate odds ratios (ORs) and 95% confidence intervals (95% CIs). Seronegative nonsmoking patients were used as the reference group. The total score of autoantibodies was calculated. Ordinal regression was used to assess the association between smoking and the number of autoantibodies.

Subsequently, a pooled analysis was performed using individual patient data from the respective cohorts. A random-effects model was used because statistical heterogeneity was present (*p* < 0.10 using the *Q* statistic) in some analyses. Correction for levels of anti-CCP2 and RF was applied using log_10_-transformed autoantibody levels because of skewness of these levels.

In the EAC and the NOAR, biological interaction between smoking and HLA SE alleles, defined as the deviation from additivity of the corresponding estimates of the outcome, was assessed by three measures: RERI, relative excess risk due to interaction; AP, the attributable proportion due to interaction; and S, the synergy index. These measures indicate a significant biological interaction if they differ from 0 (RERI and AP) or from 1 (S) [29]. To obtain the parameter estimates needed for calculating these three measures, a logistic regression model was fitted and interaction data were analyzed using Microsoft Windows Excel 2007 [30].

Antibody levels among different subgroups were compared using Mann–Whitney *U* tests. The analyses were performed per cohort using SPSS version 22.0. For the pooled analysis MedCalc software was used. *p* < 0.05 was considered statistically significant.

## Results

### Smoking and autoantibody positivity in a population-based non-RA cohort

Smoking most likely exerts its effect many years before disease development, so we first investigated the association between smoking and RA-related autoantibodies in a non-RA population-based cohort of healthy individuals: the Nagahama study.

Of the 9575 participants in this study, 35% (*n* = 3356) ever smoked, 1.7% (*n* = 167) were anti-CCP2-positive, and 5.3% (*n* = 514) were RF-positive. There was no association between smoking and the presence anti-CCP2 or RF in these healthy individuals. However, smoking was significantly associated with the presence of both of these autoantibodies (Table 1). This observation suggests that smoking may lead to the development of multiple autoantibodies, rather than one specific autoantibody.

**Table 1:** Odds ratios for RF and anti-CCP2 autoantibodies in association with smoking in a population-based non-RA cohort

	RF^–^anti-CCP2^–^ (*n* = 8836)	RF^+^anti-CCP2^–^ (*n* = 572)	RF^–^anti^-^CCP2^+^ (*n* = 125)	RF^+^anti-CCP2^+^ (*n* = 42)
Smoking ever (%)	3117 (35.3)	178 (31.1)	39 (31.2)	22 (52.4)
Smoking never (%)	5719 (64.7)	394 (68.9)	86 (68.8)	20 (47.6)
Odds ratio (95% CI)	1 (reference)	0.94 (0.75–1.18)	0.97 (0.6–1.58)	2.95 (1.32–6.58)*
*p* value	–	0.23	0.48	<0.001*

*Significant values (*p* < 0.05)

*anti-CCP2* anti-cyclic citrullinated peptide antibodies, *CI* confidence interval, *RA* rheumatoid arthritis, *RF* rheumatoid factor

### Association between smoking and autoantibody-positive RA

Next we studied the association of smoking with autoantibodies in RA patients from three independent early arthritis cohorts. The characteristics of the early arthritis cohorts are presented in Table 2. The proportion of ever smokers between the different cohorts was similar (*p* = 0.25). The prevalence of all autoantibodies was slightly lower in the NOAR compared with the EAC and the BARFOT. In all cohorts, the largest autoantibody-positive subgroup was the triple-positive subgroup.

**Table 2:** Prevalence of smoking and autoantibodies in RA patients from three different early arthritis cohorts

	NOAR	EAC	BARFOT
RA patients, *n*	678	769	795
Ever smokers	432 (64)	415 (54)	471 (59)
Anti-CarP-positive	182 (27)	349 (45)	279 (35)
Anti-CCP2-positive	247 (36)	404 (53)	456 (57)
RF-positive	277 (41)	442 (58)	448 (56)
Anti-CCP2^–^RF^–^Anti-CarP^–^	292 (43)	242 (32)	263 (33)
Anti-CCP2^+^RF^–^Anti-CarP^–^	51 (8)	20 (3)	45 (6)
Anti-CCP2^–^RF^+^Anti-CarP^–^	76 (11)	71 (9)	59 (7)
Anti-CCP2^–^RF^–^Anti-CarP^+^	40 (6)	38 (5)	6 (1)
Anti-CCP2^+^RF^+^Anti-CarP^–^	73 (11)	87 (11)	149 (19)
Anti-CCP2^+^RF^–^Anti-CarP^+^	15 (2)	27 (4)	33 (4)
Anti-CCP2^–^RF^+^Anti-CarP^+^	20 (3)	14 (2)	11 (1)
Anti-CCP2^+^RF^+^Anti-CarP^+^	107 (16)	270 (35)	229 (29)

Data presented as *n* (%) unless otherwise stated

*anti-CarP* anti-carbamylated protein antibodies, *anti-CCP2* anti-cyclic citrullinated peptide antibodies, *BARFOT* Better Anti‐Rheumatic Farmaco-Therapy, *EAC* Early Arthritis Clinic, *NOAR* Norfolk Arthritis Register, *RA* rheumatoid arthritis, *RF* rheumatoid factor

When the association between smoking and the separate autoantibodies (RF, anti-CCP2, and anti-CarP) was analyzed irrespective of the presence of other autoantibodies, a significant association was found for each autoantibody in all cohorts (Table 3).

**Table 3:** Association of smoking with anti-CCP2, RF antibodies, and anti-CarP in the RA cohorts

	Anti-CCP2^–^	Anti-CCP2^+^	RF^–^	RF^+^	Anti-CarP^–^	Anti-CarP^+^
NOAR						
*N* (total)	428	242	398	276	492	182
Ever smokers, *n* (%)	261 (61)	171 (71)	236 (59)	196 (71)	301 (61)	131 (72)
OR (95% CI)	1 (reference)	1.46 (1.04–2.03)*	1 (reference)	1.68 (1.21–2.33)*	1 (reference)	1.63 (1.12–2.36)*
*p* value	–	0.027*	–	<0.001*	–	0.01*
EAC						
*N* (total)	365	404	327	442	420	349
Ever smokers, *n* (%)	179 (49)	236 (58)	152 (47)	263 (60)	212 (51)	203 (58)
OR (95% CI)	1 (reference)	1.46 (1.10–1.94)*	1 (reference)	1.69 (1.27–2.26)*	1 (reference)	1.36 (1.03–1.82)*
*p* value	–	0.009*	–	<0.001*	–	0.03*
BARFOT						
*N* (total)	347	448	339	456	516	279
Ever smokers, *n* (%)	180 (52)	291 (65)	183 (54)	288 (63)	288 (56)	183 (66)
OR (95% CI)	1 (reference)	1.72 (1.29–2.29)*	1 (reference)	1.46 (1.01–1.95)*	1 (reference)	1.51 (1.12 – 2.04)*
*p* value	–	<0.001*	–	0.009*	–	0.008*

*Significant values (*p* < 0.05)

*anti-CarP* anti-carbamylated protein antibodies, *anti-CCP2* anti-cyclic citrullinated peptide antibodies, *BARFOT* Better Anti‐Rheumatic Farmaco-Therapy, *CI* confidence interval, *EAC* Early Arthritis Clinic, *NOAR* Norfolk Arthritis Register, *OR* odds ratio, *RA* rheumatoid arthritis, *RF* rheumatoid factor

Based on our findings in the Nagahama study, we then calculated the total number of autoantibodies per patient to investigate whether smoking may be associated with the number of autoantibodies present. The association between smoking and the number of autoantibodies is presented in Table 4, which revealed no association of smoking with either one or two autoantibodies but a significant association with triple-autoantibody positivity.

**Table 4:** Odds ratios for presence of anti-CCP2, RF autoantibodies, and anti-CarP according to smoking status

	Number of autoantibodies			
	0	1	2	3
NOAR				
*N* (total)	292	167	108	107
Smoking ever, *n* (%)	179 (61.3)	97 (58.0)	67 (62.0)	89 (83.2)
Smoking never, *n* (%)	113 (38.7)	70 (41.9)	41 (38.0)	18 (16.8)
OR (95% CI)	1 (reference)	0.87 (0.59–1.29)	1.03 (0.65–1.63)	3.12 (1.79–5.46)*
*p* value	–	0.50	0.89	<0.001*
EAC				
*N* (total)	242	129	128	270
Smoking ever, *n* (%)	113 (46.7)	67 (51.9)	70 (54.7)	165 (61.1)
Smoking never, *n* (%)	129 (53.3)	62 (48.1)	58 (45.3)	105 (38.9)
OR (95% CI)	1 (reference)	1.23 (0.80–1.89)	1.38 (0.90–2.12)	1.79 (1.26–2.55)*
*p* value	–	0.34	0.14	0.001*
BARFOT				
*N* (total)	263	110	193	229
Smoking ever, *n* (%)	138 (52.5)	58 (52.7)	121 (62.7)	154 (67.2)
Smoking never, *n* (%)	125 (47.5)	52 (47.3)	72 (37.3)	75 (32.8)
OR (95% CI)	1 (reference)	1.01 (0.65–1.58)	1.52 (1.04–2.22)*	1.86 (1.29–2.69)*
*p* value	–	0.96	0.03*	0.001*

*Significant values (*p* < 0.05)

*anti-CarP* anti-carbamylated protein antibodies, *anti-CCP2* anti-cyclic citrullinated peptide antibodies, *BARFOT* Better Anti‐Rheumatic Farmaco-Therapy, *CI* confidence interval, *EAC* Early Arthritis Clinic, *NOAR* Norfolk Arthritis Register, *OR* odds ratio, *RF* rheumatoid factor

Ordinal regression analysis showed a significant association between smoking and the number of autoantibodies in all cohorts (NOAR, *p* = 0.005; EAC, *p* = 0.001; BARFOT, *p* < 0.001), indicating a significantly stronger association in patients with many versus few autoantibodies.

**Fig. 1:**
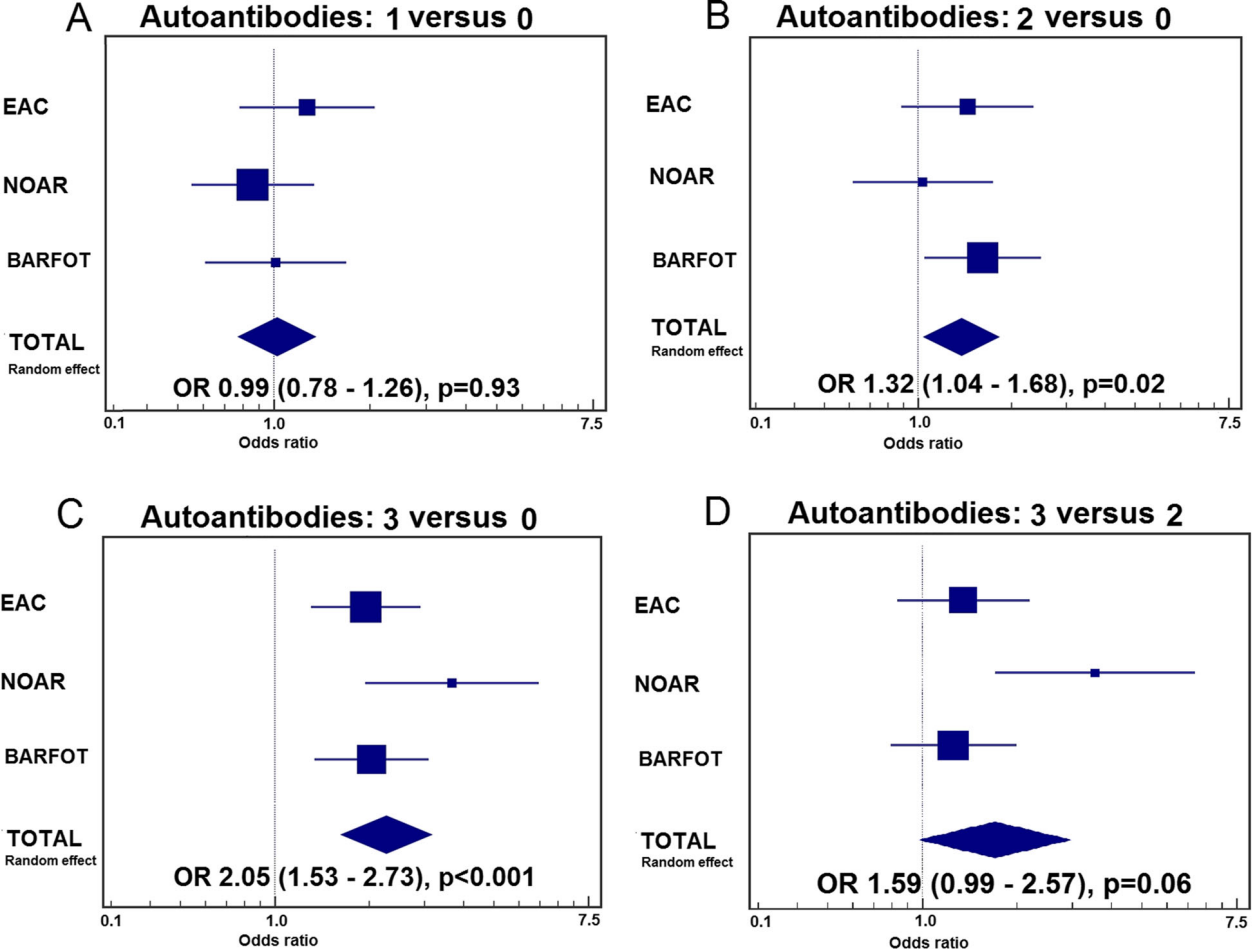
Pooled analysis investigating the association between smoking and the number of autoantibodies. Forest plots with odds ratio (*OR*) and 95% confidence interval. **a** Association of smoking with one autoantibody versus zero autoantibodies. **b** Association of smoking with two autoantibodies versus zero autoantibodies. **c** Association of smoking with three autoantibodies versus zero autoantibodies. **d** Association of smoking with three autoantibodies versus two autoantibodies. *BARFOT* Better Anti‐Rheumatic Farmaco-Therapy, *EAC* Early Arthritis Clinic, *NOAR* Norfolk Arthritis Register

Next, a pooled analysis of the three aforementioned early arthritis cohorts was performed. Although no association between ever smoking and single seropositivity could be detected (Fig. 1a), ever smoking was associated with double-autoantibody-positive RA (Fig. 1b) and triple-autoantibody-positive RA (Fig. 1c). When comparing double-positive patients with triple-positive patients, there was a trend towards a stronger association in triple-positive patients, although this did not reach statistical significance (Fig. 1d).

To see whether the association between smoking and the number of autoantibodies was caused by the increasing prevalence of one specific autoantibody among the patients with a higher autoantibody number, a subgroup analysis of all different autoantibody combinations was performed (Table 5). In the pooled analysis of the various subgroups, no significant associations were found in patients positive for one single autoantibody, be it anti-CCP2, RF, or anti-CarP. A significant association with smoking was found, however, for the anti-CCP2^+^RF^+^anti-CarP^–^ subgroup and the triple-positive subgroup compared with the triple-seronegative group. This result again suggests that smoking is associated with the presence of multiple autoantibodies rather than with a specific autoantibody. It is remarkable that compared with the seronegative RA patients, the prevalence of smoking was consistently higher in all RF-positive subgroups, but not in all anti-CCP2-positive subgroups. To investigate the added value of RF, we compared the triple-positive group with the anti-CCP2^+^RF^–^anti-CarP^+^ group. This analysis revealed a significant OR of 2.24 (95% CI 1.30–3.84). In contrast, no significant association was observed when the added value of anti-CCP2 (OR 1.18, 95% CI 0.60–2.31) or anti-CarP (OR 1.50, 95% CI 0.91–2.46) in double-positive versus triple-positive patients was analyzed. Together, these data indicate that the strong association of smoking with the triple-positive subgroup is significantly influenced by the presence of RF.

**Table 5:** Pooled analysis of the association between smoking and the presence of anti-CCP2, RF, and anti-CarP

Multicenter analysis	Anti-CCP2^–^ RF^–^anti-CarP^–^ (*n* = 797)	Anti-CCP2^+^ RF^–^anti-CarP^–^ (*n* = 116)	Anti-CCP2^–^RF^+^anti-CarP^–^ (*n* = 206)	Anti-CCP2^–^RF^–^anti-CarP^+^ (*n* = 84)	Anti-CCP2^+^RF^+^anti-CarP^–^ (*n* = 309)	Anti-CCP2^+^RF^–^anti-CarP^+^ (*n* = 75)	Anti-CCP2^–^RF^+^anti-CarP^+^ (*n* = 45)	Anti-CCP2^+^RF^+^anti-CarP^+^ (*n* = 606)
Smoking ever, *n* (%)	430 (53.9)	59 (50.9)	122 (59.2)	41 (48.8)	190 (61.5)	38 (50.7)	30 (66.7)	408 (67.3)
Smoking never, *n* (%)	367 (46.1)	57 (49.1)	84 (40.8)	43 (51.2)	119 (38.5)	37 (49.3)	15 (33.3)	198 (32.7)
OR (95% CI)	1 (reference)	0.83 (0.56–1.24)	1.25 (0.91–1.71)	0.81 (0.51–1.28)	1.40 (1.06–1.84)*	0.94 (0.58–1.51)	1.67 (0.88-3.17)	2.05 (1.53–2.73)*
*p* value	–	0.37	0.16	0.36	0.02*	0.79	0.12	<0.001*

*Significant values (*p* < 0.05)

*anti-CarP* anti-carbamylated protein antibodies, *anti-CCP2* anti-cyclic citrullinated peptide antibodies, *CI* confidence interval, *OR* odds ratio, *RF* rheumatoid factor

### Levels of autoantibodies and smoking

To examine whether the association between smoking and triple-positive RA might depend on levels of the autoantibodies, we used ordinal regression to adjust for autoantibody levels. A significant association between smoking and the number of autoantibodies was still present after correction for anti-CCP2-levels in the EAC (*p* = 0.020) and the NOAR (*p* = 0.026), but not in the BARFOT (*p* = 0.23). However, in all three cohorts the association between smoking and the number of autoantibodies was lost after correction for RF levels: EAC (*p* = 0.64), NOAR (*p* = 0.18), and BARFOT (*p* = 0.98).

### Past versus current smoking

To investigate whether the timing of smoking exposure might affect the association with RA-related autoantibodies, we performed a subgroup analysis of RA patients who smoked at the time of inclusion, called “current” smokers. The results suggested a stronger association between autoantibody positivity and current smoking than ever smoking in the NOAR (triple-positive group, OR 4.92 for current smoking versus 3.12 for ever smoking) and in the EAC (OR 2.13 versus 1.79), but not in the BARFOT (see Additional file 1 for further details). These findings suggest that the timing of smoking exposure and possibly the amount/dose of smoking may affect the relationship between smoking and autoantibodies in RA. Unfortunately, data on smoking dose in terms of pack-years were not available in all three cohorts, precluding any conclusions on a dose effect.

### Interaction analysis of smoking and SE alleles with autoantibodies

Because we found that smoking is associated with the number of autoantibodies rather than just with anti-CCP2, we then investigated whether the interaction between the HLA-DRB1 SE alleles and smoking is also dependent on the number of autoantibodies. First, the association of SE by itself with the number and different subgroups of autoantibodies was investigated in patients from the EAC and the NOAR for whom HLA typing was available. This analysis revealed an association with anti-CCP2-positive RA, but not with the number of autoantibodies, confirming previous findings (see Additional file 2 for further details) [10, 31]. Next we investigated interaction of smoking and HLA-DRB1 SE (see Additional file 3 for further details). In both cohorts, interaction was most clearly present in the triple-seropositive subgroup (with at least two of the three measures indicating significant interaction). An analysis for all subgroups of autoantibodies indicated the absence of interaction with any of the specific combinations of autoantibodies, including the ACPA-positive group, although power was limited in these subgroups. The exception was the triple-positive subgroup, in which an interaction was found (see Additional file 4 for further details).

### Smoking in relation to other autoantibodies and IgG levels

To analyze whether smoking was also associated with other autoantibodies, the association with ANA was investigated in RA patients from the EAC and non-RA individuals in the Nagahama study. No association was found between smoking and ANA positivity in both cohorts (EAC: OR 1.03, 95% CI 0.75–1.43; Nagahama: OR 0.72, 95% CI 0.63–0.82).

Finally, we examined whether smoking might enhance antibody production in general by comparing total IgG levels among ever smokers and never smokers. In RA patients from the EAC, mean serum total IgG levels did not differ between ever smokers (10.0 g/l, *n* = 174) and never smokers (10.1 g/l, *n* = 184) (*p* = 0.51).

## Discussion

This study investigated the association of smoking with the presence of several autoantibodies in RA. To our knowledge, this is the first study to investigate whether smoking is associated with a particular autoantibody or rather with the breadth of the humoral autoimmune response. The results reveal that smoking is associated with the number of RA-related autoantibodies present rather than specifically with the presence of ACPA. Smoking predisposes to the development of several autoantibodies (RF, ACPA, anti-CarP) and does not seem to affect the total amount of antibodies (total IgG) produced, but rather the variety of autoantibodies. Furthermore, the interaction between smoking and the HLA SE alleles was only present in patients with multiple autoantibodies. These data are highly relevant for current pathophysiological disease concepts, because they indicate that smoking must have a broader effect than leading only to formation of ACPA as often speculated. Smoking rather appears to contribute to the formation of an autoimmune response against several RA-associated autoantigens.

Our data are compatible with and provide an interesting new perspective on recent studies describing that, during RA disease development, smoking appears to be most important for the development of autoantibodies, whereas the contribution of the HLA SE alleles is found later in time, when they mainly affect the onset of disease in autoantibody-positive individuals. More specifically, it was shown in healthy individuals of the Nagahama study that ACPA were not associated with the presence of SE alleles [16]. Similar results were found in a Swedish study analyzing the contribution of smoking and the SE alleles to the formation of ACPA and ACPA-positive RA, suggesting that SE may have a role in determining which ACPA-positive individuals develop RA [17]. These data thus show that smoking predisposes to the development of autoantibodies, and are therefore in line with the aforementioned findings.

Our findings suggest that smoking may have a stronger association with RF than with anti-CCP or anti-CarP antibodies. In the subgroup analysis the prevalence of smoking was consistently higher in all subgroups which are RF-positive, but not in all anti-CCP2-positive subgroups or anti-CarP-positive subgroups. Furthermore, the association of smoking with number of autoantibodies was lost after correction for RF levels. The specific association between RF and smoking has also been described in studies of COPD patients, in which 42% of the patients had RF but none were ACPA-positive [32]. Similarly in mouse studies, chronic cigarette smoke was found to induce RF production in specific mice strains, but not ACPA [15]. Ultimately, it is difficult to distinguish whether smoking is associated with a higher number of autoantibodies or higher RF levels because these are highly correlated features which are both indicative of a broad, matured autoantibody response.

In our data there was no association between smoking and IgG level and the presence of ANA in RA patients. This is in line with reports that smoking is not associated with autoantibodies in other diseases, such as anti-thyroid peroxidase (TPO) antibodies in autoimmune hypothyroidism [33], and that no correlation was found between smoking and ANA in COPD patients [34]. The association between smoking and autoantibodies in systemic lupus erythematosus (SLE) is still uncertain, with some studies describing an association with anti-dsDNA antibodies, which was not replicated in other investigations [35, 36]. Altogether these observations suggest that the association of smoking with autoantibody formation in other diseases is unclear. Perhaps this association is therefore specific for RA and RA-associated autoantibodies.

There are several hypothetical pathophysiological explanations for the observed association between smoking and the simultaneous presence of various autoantibodies in RA. On the one hand, smoking may alter the autoantigens against which the immune system reacts, while on the other smoking may affect the immune system itself. For example, the metabolism of smoke substances could generate reactive oxidative species (ROS), which could lead to the modification of autoantigens and DNA adduct formation [37, 38]. Another hypothetical mechanism may involve heat shock proteins (HSPs), which have been shown to be upregulated in synovial fibroblasts of humans and mice upon smoke exposure [39]. HSPs in turn have been found to lead to production of RF in mice [15]. The interaction between smoking and genetic risk factors specific for RA may explain that smoking seems to associate only with RA-related autoantibodies.

Our study investigated the association of smoking with three autoantibodies: ACPA, RF, and anti-CarP. In our opinion, anti-CarP is a separate autoantibody, rather than a subfamily of ACPA, for two reasons. First, previous studies investigating the crossreactivity of anti-CarP and ACPA have identified partial crossreactivity, but also unique nonoverlapping specificity, as is evidenced by the presence of anti-CarP-antibodies in ACPA-negative patients [40]. Second, different risk factors have been found for ACPA and anti-CarP: ACPA is associated with SE alleles and PTPN22, whereas anti-CarP is not [21].

Limitations of this study are some heterogeneity between the cohorts with regard to patient recruitment. This probably explains part of the differences in autoantibody prevalence. In the NOAR, patient inclusion by general practitioners may have led to a milder RA phenotype with patients who are consequently less seropositive, despite their fulfillment of the 1987 RA criteria. The small patient numbers in the single autoantibody-positive groups may have led to lack of power to detect associations. However, considering the effect size close to 1.0 in the single-positive groups in the pooled analysis, it seems unlikely that a larger study population would have yielded a significant finding. Anti-CCP2 and RF were measured with different tests in the different cohorts. Literature studies have shown a high correlation among commercially available ACPA assays and different RF assays, and therefore it seems unlikely that this has affected the results [41–44]. In this study we only had data on ever smoking (split into current and past smoking) versus never smoking. Although it would have been interesting to also investigate a dose effect of smoking (e.g., by looking at pack-years), this would not have altered the direction of the association. This study was performed in European and Asian populations; therefore replication in different ethnicities is warranted. Finally, one may argue that preferably healthy controls should be used as the reference category instead of autoantibody-negative RA patients. However, although the use of healthy controls would have led to larger effect sizes for the various autoantibody subgroups, it would not have altered the relationship as a whole, because the ratio between the different autoantibody-positive subgroups would still be the same. Consequently, the strongest effect would still be present in the triple-seropositive subgroup.

Strengths of the current study include the use of a unique healthy cohort, as well as several disease cohorts, thereby capturing the entire spectrum of disease development. Furthermore, the fact that our main results were found in three independent RA cohorts indicates that these findings are very robust. Finally, the determination of a novel and promising autoantibody, anti-CarP, in these three separate cohorts enabled us to investigate the association between smoking and the number of antibodies in a more precise and refined manner than was previously possible.

## Conclusion

Smoking is not only associated with the presence of ACPA, but rather with the concurrent presence of several autoantibodies in RA. These findings shed new light on the important role of smoking in disease-specific immunomodulation in RA.

## Additional files

Additional file 1: Table presenting ORs and 95% CIs for the association of current smoking with number of autoantibodies in RA, indicating the presence of autoantibodies in current smokers versus never smokers in the different cohorts. (DOCX 15 kb)
Additional file 2: Table presenting ORs and 95% CIs for the association of SE and the number of autoantibodies in the NOAR and the EAC. (DOCX 17 kb)
Additional file 3: Table presenting biological interaction analysis between HLA-DRB1 SE alleles and smoking with the number of autoantibodies in the NOAR and the EAC. (DOCX 17 kb)
Additional file 4: Table presenting biological interaction analysis between HLA-DRB1 SE alleles and smoking for all subgroups of autoantibodies (anti-CCP2, RF, and anti-CarP) in the EAC. (DOCX 17 kb)

## Abbreviations

CI: Confidence interval
ACPA: Anti-citrullinated protein antibodies
ANA: Anti-nuclear antibodies
anti-CarP: Anti-carbamylated protein antibodies
anti-CCP2: Anti-cyclic citrullinated peptide antibodies
AP: Attributable proportion due to interaction
BARFOT: Better Anti‐Rheumatic Farmaco-Therapy
EAC: Early Arthritis Clinic
HLA: Human leukocyte antigens
HSP: Heat shock protein
Nagahama study: Nagahama Prospective Genome Cohort for Comprehensive Human Bioscience
NOAR: Norfolk Arthritis Register
OR: Odds ratios
RA: Rheumatoid arthritis
RERI: Relative excess risk due to interaction
RF: Rheumatoid factor
ROS: Reactive oxidative species
S: Synergy index
SD: Standard deviation
SE: Shared epitope
SLE: Systemic lupus erythematosus
TPO: Anti-thyroid peroxidase

## References

[1] Scott DL, Wolfe F, Huizinga TW (2010). Rheumatoid arthritis. Lancet.

[2] Silman AJ, Pearson JE (2002). Epidemiology and genetics of rheumatoid arthritis. Arthritis Res.

[3] Gregersen PK, Silver J, Winchester RJ (1987). The shared epitope hypothesis. An approach to understanding the molecular genetics of susceptibility to rheumatoid arthritis. Arthritis Rheum.

[4] van der Woude D, Rantapaa-Dahlqvist S, Ioan-Facsinay A, Onnekink C, Schwarte CM, Verpoort KN (2010). Epitope spreading of the anti-citrullinated protein antibody response occurs before disease onset and is associated with the disease course of early arthritis. Ann Rheum Dis.

[5] Gan RW, Trouw LA, Shi J, Toes RE, Huizinga TW, Demoruelle MK (2015). Anti-carbamylated protein antibodies are present prior to rheumatoid arthritis and are associated with its future diagnosis. J Rheumatol.

[6] Symmons DP, Bankhead CR, Harrison BJ, Brennan P, Barrett EM, Scott DG (1997). Blood transfusion, smoking, and obesity as risk factors for the development of rheumatoid arthritis: results from a primary care-based incident case-control study in Norfolk, England. Arthritis Rheum.

[7] Stolt P, Bengtsson C, Nordmark B, Lindblad S, Lundberg I, Klareskog L (2003). Quantification of the influence of cigarette smoking on rheumatoid arthritis: results from a population based case-control study, using incident cases. Ann Rheum Dis.

[8] Silman AJ, Newman J, MacGregor AJ (1996). Cigarette smoking increases the risk of rheumatoid arthritis. Results from a nationwide study of disease-discordant twins. Arthritis Rheum.

[9] Schellekens GA, de Jong BA, van den Hoogen FH, van de Putte LB, van Venrooij WJ (1998). Citrulline is an essential constituent of antigenic determinants recognized by rheumatoid arthritis-specific autoantibodies. J Clin Invest.

[10] Huizinga TW, Amos CI, van der Helm-van Mil AH, Chen W, van Gaalen FA, Jawaheer D (2005). Refining the complex rheumatoid arthritis phenotype based on specificity of the HLA-DRB1 shared epitope for antibodies to citrullinated proteins. Arthritis Rheum.

[11] Willemze A, Trouw LA, Toes RE, Huizinga TW (2012). The influence of ACPA status and characteristics on the course of RA. Nat Rev Rheumatol.

[12] Kallberg H, Padyukov L, Plenge RM, Ronnelid J, Gregersen PK, van der Helm-van Mil AH (2007). Gene-gene and gene-environment interactions involving HLA-DRB1, PTPN22, and smoking in two subsets of rheumatoid arthritis. Am J Hum Genet.

[13] Linn-Rasker SP, van der Helm-van Mil AH, van Gaalen FA, Kloppenburg M, de Vries RR, le Cessie S (2006). Smoking is a risk factor for anti-CCP antibodies only in rheumatoid arthritis patients who carry HLA-DRB1 shared epitope alleles. Ann Rheum Dis.

[14] Klareskog L, Stolt P, Lundberg K, Källberg H, Bengtsson C, Grunewald J (2006). A new model for an etiology of rheumatoid arthritis: smoking may trigger HLA-DR (shared epitope)-restricted immune reactions to autoantigens modified by citrullination. Arthritis Rheum.

[15] Newkirk MM, Mitchell S, Procino M, Li Z, Cosio M, Mazur W (2012). Chronic smoke exposure induces rheumatoid factor and anti-heat shock protein 70 autoantibodies in susceptible mice and humans with lung disease. Eur J Immunol.

[16] Terao C, Ohmura K, Ikari K, Kawaguchi T, Takahashi M, Setoh K (2014). Effects of smoking and shared epitope on the production of anti-citrullinated peptide antibody in a Japanese adult population. Arthritis Care Res (Hoboken).

[17] Hensvold AH, Magnusson PK, Joshua V, Hansson M, Israelsson L, Ferreira R (2015). Environmental and genetic factors in the development of anticitrullinated protein antibodies (ACPAs) and ACPA-positive rheumatoid arthritis: an epidemiological investigation in twins. Ann Rheum Dis.

[18] Shi J, Knevel R, Suwannalai P, van der Linden MP, Janssen GM, van Veelen PA (2011). Autoantibodies recognizing carbamylated proteins are present in sera of patients with rheumatoid arthritis and predict joint damage. Proc Natl Acad Sci U S A.

[19] Shi J, van de Stadt LA, Levarht EW, Huizinga TW, Toes RE, Trouw LA (2013). Anti-carbamylated protein antibodies are present in arthralgia patients and predict the development of rheumatoid arthritis. Arthritis Rheum.

[20] Shi J, van de Stadt LA, Levarht EW, Huizinga TW, Hamann D, van Schaardenburg D (2014). Anti-carbamylated protein (anti-CarP) antibodies precede the onset of rheumatoid arthritis. Ann Rheum Dis.

[21] Jiang X, Trouw LA, van Wesemael TJ, Shi J, Bengtsson C, Kallberg H (2014). Anti-CarP antibodies in two large cohorts of patients with rheumatoid arthritis and their relationship to genetic risk factors, cigarette smoking and other autoantibodies. Ann Rheum Dis.

[22] Brink M, Verheul MK, Ronnelid J, Berglin E, Holmdahl R, Toes RE (2015). Anti-carbamylated protein antibodies in the pre-symptomatic phase of rheumatoid arthritis, their relationship with multiple anti-citrulline peptide antibodies and association with radiological damage. Arthritis Res Ther.

[23] Humphreys JH, Verheul MK, Barton A, MacGregor AJ, Lunt M, Toes RE (2015). Anticarbamylated protein antibodies are associated with long-term disability and increased disease activity in patients with early inflammatory arthritis: results from the Norfolk Arthritis Register. Ann Rheum Dis.

[24] Symmons DP, Barrett EM, Bankhead CR, Scott DG, Silman AJ (1994). The incidence of rheumatoid arthritis in the United Kingdom: results from the Norfolk Arthritis Register. Br J Rheumatol.

[25] De Rooy DP, van der Linden MP, Knevel R, Huizinga TW, van der Helm-van Mil AH (2011). Predicting arthritis outcomes—what can be learned from the Leiden Early Arthritis Clinic?. Rheumatol (Oxford).

[26] Svensson B, Ahlmen M, Forslind K (2003). Treatment of early RA in clinical practice: a comparative study of two different DMARD/corticosteroid options. Clin Exp Rheumatol.

[27] van der Horst-Bruinsma IE, Visser H, Hazes JM, Breedveld FC, Verduyn W, Schreuder GM (1999). HLA-DQ-associated predisposition to and dominant HLA-DR-associated protection against rheumatoid arthritis. Hum Immunol.

[28] Terao C, Ohmura K, Yamada R, Kawaguchi T, Shimizu M, Tabara Y (2014). Association between antinuclear antibodies and the HLA class II locus and heterogeneous characteristics of staining patterns: the Nagahama study. Arthritis Rheumatol.

[29] Rothman KJ (2002). Measuring Interactions. Epidemiology: An Introduction.

[30] Andersson T, Alfredsson L, Kallberg H, Zdravkovic S, Ahlbom A (2005). Calculating measures of biological interaction. Eur J Epidemiol.

[31] Irigoyen P, Lee AT, Wener MH, Li W, Kern M, Batliwalla F (2005). Regulation of anti-cyclic citrullinated peptide antibodies in rheumatoid arthritis: contrasting effects of HLA-DR3 and the shared epitope alleles. Arthritis Rheum.

[32] Yang DH, Tu CC, Wang SC, Wei CC, Cheng YW (2014). Circulating anti-cyclic citrullinated peptide antibody in patients with rheumatoid arthritis and chronic obstructive pulmonary disease. Rheumatol Int.

[33] Pedersen IB, Laurberg P, Knudsen N, Jorgensen T, Perrild H, Ovesen L (2008). Smoking is negatively associated with the presence of thyroglobulin autoantibody and to a lesser degree with thyroid peroxidase autoantibody in serum: a population study. Eur J Endocrinol.

[34] Feghali-Bostwick CA, Gadgil AS, Otterbein LE, Pilewski JM, Stoner MW, Csizmadia E (2008). Autoantibodies in patients with chronic obstructive pulmonary disease. Am J Respir Crit Care Med.

[35] Young KA, Terrell DR, Guthridge JM, Kamen DL, Gilkeson GS, Karp DR (2014). Smoking is not associated with autoantibody production in systemic lupus erythematosus patients, unaffected first-degree relatives, nor healthy controls. Lupus.

[36] Freemer MM, King TE, Criswell LA (2006). Association of smoking with dsDNA autoantibody production in systemic lupus erythematosus. Ann Rheum Dis.

[37] Petruzzelli S, Celi A, Pulera N, Baliva F, Viegi G, Carrozzi L (1998). Serum antibodies to benzo(a)pyrene diol epoxide-DNA adducts in the general population: effects of air pollution, tobacco smoking, and family history of lung diseases. Cancer Res.

[38] Mooney LA, Perera FP, Van Bennekum AM, Blaner WS, Karkoszka J, Covey L (2001). Gender differences in autoantibodies to oxidative DNA base damage in cigarette smokers. Cancer Epidemiol Biomarkers Prev.

[39] Ospelt C, Camici GG, Engler A, Kolling C, Vogetseder A, Gay RE (2014). Smoking induces transcription of the heat shock protein system in the joints. Ann Rheum Dis.

[40] Shi J, Willemze A, Janssen GM, van Veelen PA, Drijfhout JW, Cerami A (2013). Recognition of citrullinated and carbamylated proteins by human antibodies: specificity, cross-reactivity and the ‘AMC-Senshu’ method. Ann Rheum Dis.

[41] Aggarwal R, Liao K, Nair R, Ringold S, Costenbader KH (2009). Anti-citrullinated peptide antibody assays and their role in the diagnosis of rheumatoid arthritis. Arthritis Rheum.

[42] Coenen D, Verschueren P, Westhovens R, Bossuyt X (2007). Technical and diagnostic performance of 6 assays for the measurement of citrullinated protein/peptide antibodies in the diagnosis of rheumatoid arthritis. Clin Chem.

[43] Bampton JL, Cawston TE, Kyle MV, Hazleman BL (1985). Measurement of rheumatoid factors by an enzyme-linked immunosorbent assay (ELISA) and comparison with other methods. Ann Rheum Dis.

[44] Jaspers JP, Van Oers RJ, Leerkes B (1988). Nine rheumatoid factor assays compared. J Clin Chem Clin Biochem.

