# Supplementary material for: Smoking is associated with the concurrent presence of multiple autoantibodies in rheumatoid arthritis rather than with anti-citrullinated protein antibodies per se: a multicenter cohort study
Source: Arthritis Res Ther. 2016 Dec 1;18:285. doi: 10.1186/s13075-016-1177-9 (PMC5134292; doi:10.1186/s13075-016-1177-9)
Supplement: Additional file 1: — Table presenting ORs and 95% CIs for the association of current smoking with number of autoantibodies in RA, indicating the presence of autoantibodies in current smokers versus never smokers in the different cohorts. (DOCX 15 kb) [file 13075_2016_1177_MOESM1_ESM.docx]

Additional file 1. Odds ratio’s for presence of autoantibodies in current smokers versus never smokers in the RA-cohorts

| Number of autoantibodies | 0 | 1 | 2 | 3 |
| --- | --- | --- | --- | --- |
| **NOAR** | | | | |
| N total | 171 | 103 | 65 | 60 |
| Smoking current (%) | 55 (32.2) | 33 (32.0) | 23 (35.4) | 42 (70.0) |
| Smoking never (%) | 116 (67.8) | 70 (68.0) | 42 (64.6) | 18 (30.0) |
| OR (95% CI) | 1 (ref) | 0.99 (0.59-1.68) | 1.15 (0.63-2.11) | **4.92 (2.60-9.32)** |
| p-value | - | 0.98 | 0.64 | **<0.001** |
| **EAC** | | | | |
| N total | 182 | 94 | 98 | 197 |
| Smoking current (%) | 53 (29.1) | 32 (34.0) | 40 (40.8) | 92 (46.7) |
| Smoking never (%) | 129 (70.9) | 62 (66.0) | 58 (59.2) | 105 (53.3) |
| OR (95% CI) | 1 (ref) | 1.26 (0.74 – 2.14) | **1.68 (1.00 – 2.81)** | **2.13 (1.39 – 3.26)** |
| p-value | - | 0.40 | **0.05** | **<0.001** |
| **BARFOT** | | | | |
| N total | 190 | 73 | 136 | 161 |
| Smoking current (%) | 65 (34.2) | 21 (28.8) | 64 (47.1) | 86 (53.4) |
| Smoking never (%) | 125 (65.8) | 52 (71.2) | 72 (52.9) | 75 (46.6) |
| OR (95% CI) | 1 (ref) | 0.88 (0.66 – 1.18) | **1.31 (1.04 – 1.64)** | **1.49 (1.20 – 1.84)** |
| p-value | - | 0.40 | **0.02** | **<0.001** |

Bold print indicates significant values (p< 0.05).
