# Supplementary material for: Smoking is associated with the concurrent presence of multiple autoantibodies in rheumatoid arthritis rather than with anti-citrullinated protein antibodies per se: a multicenter cohort study
Source: Arthritis Res Ther. 2016 Dec 1;18:285. doi: 10.1186/s13075-016-1177-9 (PMC5134292; doi:10.1186/s13075-016-1177-9)
Supplement: Additional file 2: — Table presenting ORs and 95% CIs for the association of SE and the number of autoantibodies in the NOAR and the EAC. (DOCX 17 kb) [file 13075_2016_1177_MOESM2_ESM.docx]

Additional file 2: The association of SE and number of autoantibodies in the NOAR and EAC

| Number of autoantibodies | 0 | 1 | *CCP2+RF-CarP-* | *CCP2-RF+CarP-* | *CCP2-RF-CarP+* | 2 | *CCP2+RF+CarP-* | *CCP2+RF-CarP+* | *CCP2-RF+CarP+* | 3 |
| --- | --- | --- | --- | --- | --- | --- | --- | --- | --- | --- |
| **NOAR** | | | | | | | | | | |
| N total | 244 | 132 | *44* | *57* | *31* | 81 | *55* | *12* | *14* | 87 |
| SE pos, n(%) | 106 (43) | 82(62) | *35(80)* | *32(56)* | *15(48)* | 55(68) | *41(75)* | *9(75)* | *5(36)* | 72 (83) |
| SE neg, n(%) | 138 (57) | 50(38) | *9(21)* | *25(44)* | *16(52)* | 26(32) | *14(25)* | *3(25)* | *9(64)* | 15(17) |
| OR (95% CI) | 1 (ref) | **2.13 (1.38-3.29)** | ***5.06 (2.33-11.00)*** | *1.67 (0.93-2.98)* | *1.22 (0.58-2.58)* | **2.75 (1.62-4.68)** | ***3.81(1.98-7.38)*** | ***3.91(1.03-14.78)*** | *0.72(0.26-2.22)* | **6.24 (3.39-11.51)** |
| p-value | - | **0.001** | ***<0.001*** | *0.09* | *0.60* | **<0.001** | ***<0.001*** | ***0.045*** | *0.57* | **<0.001** |
| **EAC** | | | | | | | | | | |
| N total | 204 | 109 | *16* | *60* | *33* | 103 | *73* | *22* | *8* | 87 |
| SE pos, n(%) | 102 (50) | 55 (50) | *15 (94)* | *25 (42)* | *15 (45)* | 75 (72.8) | *54 (74)* | *19 (86)* | *2 (25)* | 0.72(0.26-2.22) |
| SE neg, n(%) | 102 (50) | 54 (50) | *1 (6)* | *35 (58)* | *18(55)* | 28 (27.2) | *19 (26)* | *3(25)* | *6 (75)* | 15(17) |
| OR (95% CI) | 1 (ref) | 1.02 (0.64 – 1.62) | ***15.00 (1.95 – 115.68)*** | *0.71 (0.40 – 1.28)* | *0.82 (0.40 – 1.74)* | **2.70 (1.60 - 4.48)** | ***2.84 (1.58 – 5.13)*** | ***6.33 (1.82 – 22.07)*** | *0.72(0.26-2.22)* | **6.24 (3.39-11.51)** |
| p-value | - | 0.94 | ***0.009*** | *0.26* | *0.63* | **<0.001** | ***0.001*** | ***0.004*** | *0.57* | **<0.001** |

Bold print indicates significant values (p< 0.05).
