# Supplementary material for: Smoking is associated with the concurrent presence of multiple autoantibodies in rheumatoid arthritis rather than with anti-citrullinated protein antibodies per se: a multicenter cohort study
Source: Arthritis Res Ther. 2016 Dec 1;18:285. doi: 10.1186/s13075-016-1177-9 (PMC5134292; doi:10.1186/s13075-016-1177-9)
Supplement: Additional file 3: — Table presenting biological interaction analysis between HLA-DRB1 SE alleles and smoking with the number of autoantibodies in the NOAR and the EAC. (DOCX 17 kb) [file 13075_2016_1177_MOESM3_ESM.docx]

Additional file 3. Biological interaction analysis between HLA-DRB1 SE alleles and smoking with number of autoantibodies in the EAC and NOAR in RA

|  | RERI | AP | S |
| --- | --- | --- | --- |
| NOAR, n=544 |  |  |  |
| 0 ab | (referent) | (referent) | (referent) |
| 1 ab | -0.889 (-2.580-0.802) | -0.542 (-1.551-0.467) | 0.419 (0.17-1.499) |
| 2 ab | 0.240 (-1.962-2.406) | 0.083 (-0.66-0.825) | 1.145 (0.313-0.419) |
| 3 ab | 11.98 (-2.372-26.331) | **0.706 (0.484-0.929)** | **4.012 (1.459-11.034)** |
| EAC, n=652 |  |  |  |
| 0 ab | (referent) | (referent) | (referent) |
| 1 ab | -0.65 (-2.96 – 1.65) | -0.21 (-0.99 – 0.57) | 0.77 (0.31 – 1.89) |
| 2 abs | 42.94 (-0.45 – 86.33) | **0.57 (0.34 – 0.81)** | **2.39 (1.35 – 4.23)** |
| 3 abs | **357.58 (143.52 – 571.63)** | **0.95 (0.92 – 0.97)** | **19.37 (12.01 – 31.25)** |

The biological interaction measures indicate a significant interaction if they differ from 0 (RERI and AP) or 1 (S).
