# Supplementary material for: Smoking is associated with the concurrent presence of multiple autoantibodies in rheumatoid arthritis rather than with anti-citrullinated protein antibodies per se: a multicenter cohort study
Source: Arthritis Res Ther. 2016 Dec 1;18:285. doi: 10.1186/s13075-016-1177-9 (PMC5134292; doi:10.1186/s13075-016-1177-9)
Supplement: Additional file 4: — Table presenting biological interaction analysis between HLA-DRB1 SE alleles and smoking for all subgroups of autoantibodies (anti-CCP2, RF, and anti-CarP) in the EAC. (DOCX 17 kb) [file 13075_2016_1177_MOESM4_ESM.docx]

Additional file 4. Biological interaction analysis between HLA-DRB1 SE alleles and smoking for all subgroups of autoantibodies in the EAC in RA

|  | RERI | AP | S |
| --- | --- | --- | --- |
| EAC, n=652 |  |  |  |
| 0 ab | (referent) | (referent) | (referent) |
| 1 ab | -0.65 (-2.96 – 1.65) | -0.21 (-0.99 – 0.57) | 0.77 (0.31 – 1.89) |
| *Anti-CCP2+RF-Anti-CarP-* | -6622.78 (-9807.21 – -3438.35) | -4.47 (-16.26 – 7.31) | 0.18 (0.02 – 1.57) |
| *Anti-CCP2-RF+Anti-CarP-* | **2.03 (0.06 – 4.00)** | **0.68 (0.28 – 1.08)** | - |
| *Anti-CCP2-RF-Anti-CarP+* | -1.49 (-4.79 – 1.80) | -0.70 (-2.73 – 1.19) | 0.39 (0.05 – 2.87) |
| 2 abs | 42.94 (-0.45 – 86.33) | **0.57 (0.34 – 0.81)** | **2.39 (1.35 – 4.23)** |
| *Anti-CCP2+RF+Anti-CarP-* | 204.09 (-18.32 – 426.49) | **0.70 (0.51 – 0.89)** | **3.32 (1.77 – 6.19)** |
| *Anti-CCP2+RF-Anti-CarP+* | 349.24 (-230.68 – 929.17) | **0.79 (0.59 – 1.00)** | **4.89 (1.81 – 13.23)** |
| *Anti-CCP2-RF+Anti-CarP+* | -2.27 (-9.09 – 4.54) | -1.51 (-7.49 – 4.46) | 0.18 (0.00 – 104.61) |
| 3 abs | **357.58 (143.52 – 571.63)** | **0.95 (0.92 – 0.97)** | **19.37 (12.01 – 31.25)** |

The biological interaction measures indicate a significant interaction if they differ from 0 (RERI and AP) or 1 (S).
